# Properties of Surface Heating Textile for Functional Warm Clothing Based on a Composite Heating Element with a Positive Temperature Coefficient

**DOI:** 10.3390/nano11040904

**Published:** 2021-04-01

**Authors:** Han Na Choi, Seung Hyun Jee, Jaehwan Ko, Dong Joo Kim, Sun Hee Kim

**Affiliations:** 1 Department of Clothing & Textiles, Kyunghee University, Seoul 02447, Korea; hanna4102@gmail.com; 2 Mcell Co., Ltd., 407, 815, Daewangpangyo-ro, Sujeong-gu, Seongnam-si, Gyeonggi-do 13449, Korea; jsh@mcell.co.kr; 3 Department of Materials Science and Engineering, Gachon University, Seongnam 13120, Korea; jhko1775@gmail.com; 4 Materials Research and Education Center, Auburn University, Auburn, AL 36849, USA; 5 Department of Fashion Industry, Incheon University, Incheon 22012, Korea

**Keywords:** surface heating elements, IC joule heater, heating performance, heating textile, heated clothing, CNT

## Abstract

A high-stretch positive temperature coefficient (PTC) surface heating textile (PTC-SHT) was fabricated using a composite of PTC powder and multiwall carbon nanotubes (MWCNTs). The PTC-SHT (heating area = 100 × 100 mm^2^) was produced by screen-printing the PTC-MWCNT composite paste onto a high-stretch textile with embroidered electrodes. Overall, the temperature increased to 56.1 °C with a power consumption of 5 W over 7 min. Subsequently, the surface temperature of the PTC-SHT remained constant despite the continued decrease in power consumption. This indicated that heating was accompanied by an increase in resistance of the PTC-SHT, which is typical of this process—i.e., heating to a constant temperature under a constant voltage over an extended period of time. In addition, 4.63 W power was required to heat the PTC-SHT surface from an external temperature of 5 to 45 °C in 10 min, after which stable low-temperature heat generation behavior was observed at a constant temperature of 50 °C, which was maintained over 40 min. In contrast, negative temperature coefficient (NTC) behavior has been observed in an NTC-SHT consisting of only MWCNTs, where a slow heating rate in the initial stage of power application and a continuous increase in surface temperature and power consumption were noted. The PTC-SHT consumed less power for heat generation than the NTC-SHT and exhibited rapid heating behavior in the initial stage of power application. The heat generation characteristics of the PTC-SHT were maintained at 95% after 100,000 cycles of 20% stretch–contraction testing, and the heating temperature remained uniformly distributed within ± 2 °C across the entire heating element. These findings demonstrated that an SHT with PTC characteristics is highly suitable for functional warm clothing applications that require low power consumption, rapid heating, stable warmth, and high durability.

## 1. Introduction

Functional clothing is described as the incorporation of information technology (IT) in conventional clothing, where research interest regarding the related technologies and products is growing rapidly. Specifically, heated clothing has been previously developed for aerospace, combat suits, and other specialized applications. However, reductions in the costs associated with this technology have shifted the focus of research and development to heated clothing for everyday use [1,2,3,4,5]. This rise in popularity of heated clothing is attributed to its auxiliary functions for outdoor and daily life, and also for medical purposes such as promoting blood circulation and alleviation of muscle pain and arthritis. Heating element technology is an important aspect of heated clothing development and manufacturing, where durability, stable heat generation, and low power consumption during use are crucial factors. Heat generation for clothing with warming functionalities can be achieved using various methods, where surface heating, conductive textile heating, wire heating, metal heating, and conductive ink printing are the most actively studied. However, durability issues have led to the simultaneous development of detachable heating elements and heat control devices [6,7,8,9,10].

Metal and carbon-based heating element materials are the most popular in heated clothing and are typically used for linear heating as heating wires. However, heating wires are susceptible to short circuiting, particularly due to folding of the materials, and although highly flexible metal heating wires capable of rapid heat generation are used, issues such as uneven heat distribution and inconvenience when wearing remain. Ceramic powder-based heating materials have been recently reported for application in heated clothing [11]. Surface heating is a promising alternative to overcome the disadvantages associated with linear heating. However, a negative temperature coefficient (NTC) is typically observed in these materials, where the internal resistance decreases as the temperature of the heating element increases. This can lead to power supply control issues [12]. In response, a previous study reported a planar heating element with a positive temperature coefficient (PTC), where the resistance of the heating element increased with increasing temperature. A large current was applied at a low temperature, and the low resistance facilitated rapid heating within a short time. In addition, heat balance between heat generation and dissipation was achieved at high temperatures by reducing heat generation, which allowed for a constant temperature to be maintained [13,14,15]. PTC characteristics have proven to be useful in various applications, such as controlling the fuel temperature of automobile engines and defrosting heat pump systems [16,17,18,19].

**Figure 1:**
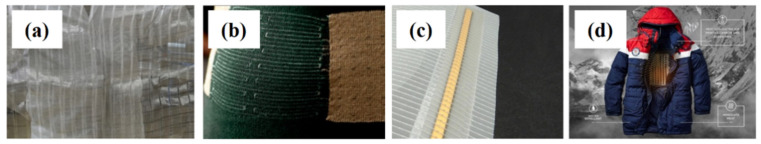
Examples of textiles based on heating elements with metal wires and Surface heating elements (SHEs): (**a**) a metal heating element, (**b**) Warm X, (**c**) SEFAR Power Heat, and (**d**) novel outdoor warm clothing.

Surface heating elements (SHEs) have been used in various applications (Figure 1). For example, functional clothing such as thermal jackets and heated jackets with active warming functions have been recently commercialized. In particular, Warm X and SEFAR Power Heat have launched functional warm clothing with improved fit by using novel textile manufacturing techniques such as knitting and embroidery [20]. SEFAR power heat has a polyester monofilament mesh structure and is capable of rapid heating and maintaining thermal insulation in case of cuts or other partial damage.

Microfiber heating blankets, graphene-based heating bedding, and flexible flat heating elements based on stainless steel metal fiber electrodes are commercially available, and the application range of this technology continues to expand. However, warm clothing with full thermostatic properties at specific temperatures has not yet been reported [21,22,23,24,25,26]. A heating element without PTC characteristics relies on a thermostat or electronic circuit to control the applied power, but these components can be associated with discomfort during wearing or activity, performance degradation after washing, and burn or fire risks due to abnormal heat generation. Instead, the effective implementation of functional warm clothing relies on the development of surface heating textiles (SHTs), which offer high elasticity and warmth, rapid heating to a set temperature, and heat generation at the desired level with low power consumption. Further, such SHTs must have high durability for washing and continued use.

This study developed an SHT with PTC heating characteristics (PTC-SHT) based on a composite paste comprising mechanically mixed multiwalled carbon nanotubes (MWCNTs) and a commercial PTC paste. The PTC-MWCNT composite paste was screen-printed on a highly stretchable textile with embroidered electrodes, and the PTC coefficient and microstructure of the PTC-SHT were experimentally evaluated according to various heating element manufacturing conditions. The effect of PTC on the characteristics of the SHT was evaluated by comparing the heating characteristics according to applied voltage, power consumption, and operating time with a negative temperature coefficient (NTC) SHT (NTC-SHT) comprising only MWCNTs. Further, the durability of the PTC-SHT during repeated stretching–contraction and washing cycles was determined to demonstrate its application potential in functional warm clothing.

## 2. Experimental

### 2.1. Composite PTC Paste Preparation

**Figure 2:**
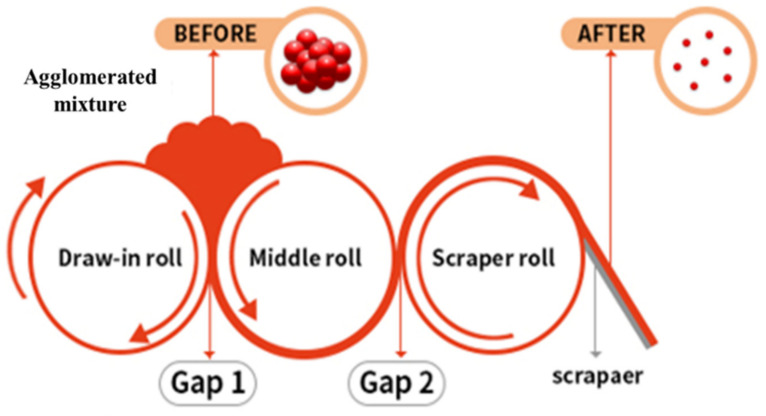
Three-roll milling of agglomerated mixture for refinement and uniformly dispersion.

Screen-printing of composite materials relies on the preparation of an optimal paste [27,28]. A composite paste with PTC behavior was prepared by mechanically mixing commercially available PTC carbon ink (Ink−1M7511, 1-Material) and MWCNT (7311, 1-Material) powders. The PTC ink facilitated the preparation of a paste without the addition of a binder. MWCNT powder (0.05 or 0.06 g) was mixed with the PTC ink (20 g) in a 200 mL PVC mixing container to give pastes with MWCNT contents of 0.25 and 0.3 wt%. The PTC ink and MWCNT powder were mixed for 3 min at a rotation speed of 1500 rpm and a revolution speed of 150 rpm using a planetary mixer, where mixing was performed twice to ensure uniform mixing and defoaming. The agglomerated mixture was refined by passing the mixture through a three-roll mill (EXAKT 50) five times, where gaps 1 and 2 were 15 and 7.5 µm in the first pass, respectively, 10 and 5 µm in the second pass, 5 and 2.5 um in the third pass, 3 and 1.5 in the fourth pass, and 1 and 1µm in the fifth pass (Figure 2).

### 2.2. Fabrication of PTC-SHT and NTC-SHT

**Figure 3:**
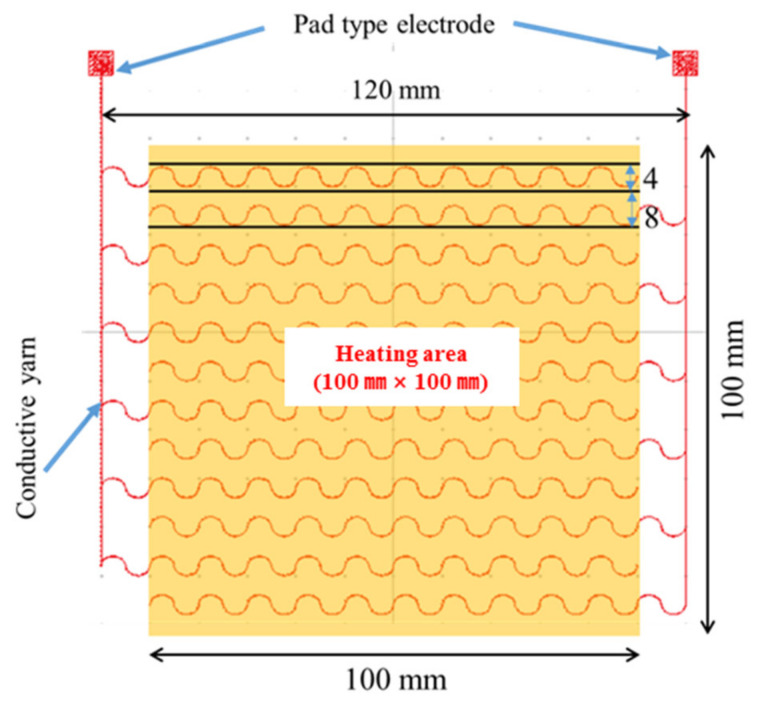
Embroidered electrodes with a sine wave pattern for power supply of the SHE.

The PTC paste was screen-printed on a high-stretch fabric (86% polyester, 14% polyurethane; 70D SHT, Chungnam Textile) with electrodes of embroidered conductive yarn to supply powder for heating functionality. The electrode pattern was formed via computer embroidery (PE-Design 1.0 software, BROTHER CO., LTD, Seoul, Korea) of conductive yarn (diameter = 0.5 mm) comprising three strands of Ag string and three strands of polyester string (Figure 3). Twelve lines of embroidery were used to prevent damage to the electrode during stretching of the high-stretch fabric, where the form of each line was a sine wave with an amplitude of 4 mm. The lines were spaced at 8 mm intervals. Even power supply of the entire electrode line was achieved by connecting six odd lines (+) and six even lines (–) in parallel, where two pad-type electrodes were placed outside the SHE area to connect the (+) and (–) lines.

The composite PTC paste was screen-printed twice using a 50-mesh screen. The first screen-printing cycle was performed at a pressure of 0.2 kg/cm^2^ to ensure sufficient immersion of the PTC paste inside the fabric with embroidered electrodes. The screen-printed fabric was air-dried for 5 min and hot-dried in an oven at 130 °C for 5 min. The second screen-printing cycle was performed at a lower pressure of 0.1 kg/cm^2^ to prevent cracking or peeling of the PTC paste layer from the first cycle. The subsequent drying and curing were performed simultaneously in a 130 °C oven for 20 min without air drying.

An eyelet electrode was formed on each of the two electrode pads, and the final SHE was soldered to an external power line. The eyelet electrode minimized the contact resistance between the power wiring and electrode pad, thereby reducing the consumption of applied power in the electrode pad caused by resistance heat. Thus, the SHE consumed all of the applied power.

**Figure 4:**
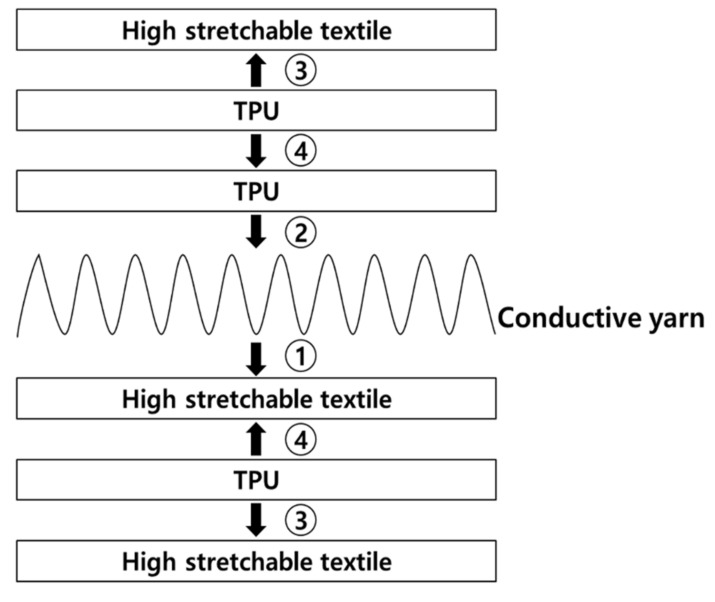
Positive temperature coefficient surface heating textile (PTC-SHT) assembly conducted in four steps. ① electrode patterning and screen printing, ② TPU film bonding, ③ External textile preparation (TPU film bonding), ④ Bonding of inner and outer textiles.

**Figure 5:**
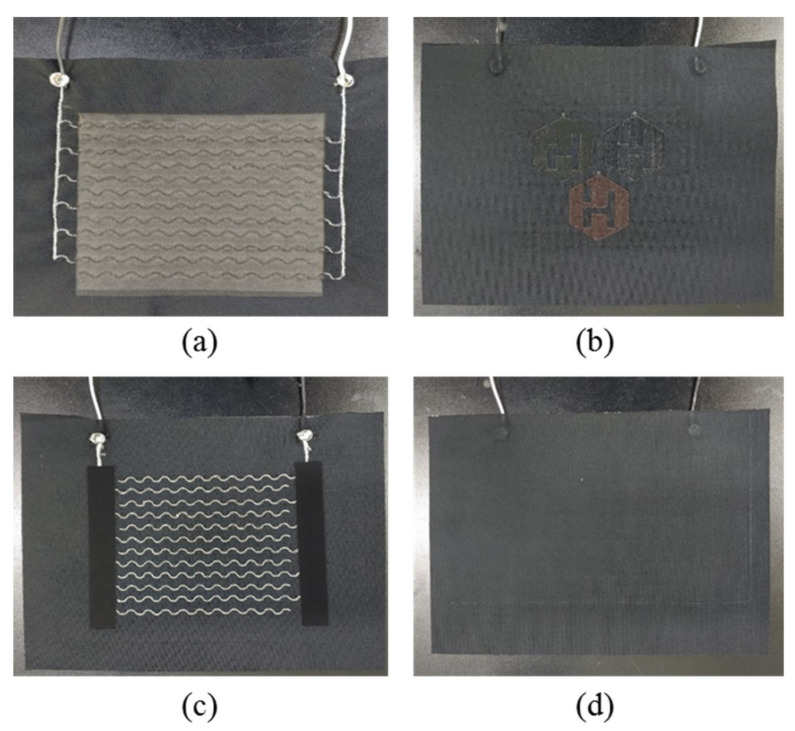
Digital photographs of the front: (**a**) after screen-printing; (**b**) finished product and back (**c**) after screen-printing; (**d**) finished product of the SHT.

A sealing structure was formed on the final PTC-SHT to electrically insulate the SHE and minimize damage due to external physical factors (Figure 4). A thermoplastic polyurethane (TPU) film (thickness = 150 μm; Ceylon 3095) was applied on top of the prepared SHE fabric via thermal transfer (165 °C for 15 s under a load of 10 kg) (Figure 4, step②). Two sections of high-stretch fabric with the same dimensions were prepared separately, where TPU was also applied to one surface via thermal transfer (Figure 4, step③). The two fabric sections were attached to the top and bottom of the SHE by thermally bonding the TPU-coated fabric surfaces (Figure 4, step④). The final PTC-SHT comprised three materials—namely, three layers of high-stretch fabric (0.4 mm × 3), one SHE (0.2 mm), and three TPU adhesive films (0.15 mm × 2), where the total thickness was determined as 1.7 mm using a Vernier caliper. The final PTC-SHT was easily folded akin to a regular fabric with a similar thickness (Figure 5).

A self-produced NTC-SHT with typical NTC properties was prepared to serve as comparison for the evaluation of the PTC-SHT. The NTC-SHE was fabricated using an aqueous paste containing only MWCNTs as the heating component. Hydroxypropyl methyl cellulose (HPMC) with a molecular weight of 86,000 was used as a binder for the aqueous paste. A solution with a viscosity of 73,000 cP was prepared by stirring HPMC (20 g) in 400 mL of distilled water (specific resistance >10^6^ Ω-cm/cm). MWCNTs (16 g) were added to the HPMC solution and ultrasonically mixed for 1 h. The paste was screen-printed four times on the high-stretch fabric using a 50-mesh screen over an area of 130 × 77 mm^2^ (within 0.1% difference from the 100 × 100 mm^2^ PTC-SHE area). The NTC-SHE was dried in an oven at 130 °C for 5 min after the first, second and, third screen-printing cycles, and cured for 20 min at the same temperature after the fourth. Sections of high-stretch elastic fabric with thermal transfer TPU were attached to the top and bottom of the SHE to produce an NTC-SHT via the same procedure used for the PTC-SHT. No visible cracks were observed on the surface of the NTC-SHT. The behavior of the PTC-SHT and NTC-SHT heating elements were compared based on the applied voltage, applied power, and heating temperature change under a constant voltage.

### 2.3. Characterization of the PTC-SHT

#### 2.3.1. Surface Morphology

The surface of the PTC-SHE screen-printed twice with 0.25 wt% MWCNTs was imaged using scanning electron microscopy (SEM, Hitachi, MARUNOCHI, Japan).

#### 2.3.2. Resistance and Resistance Ratio

The resistivity of the SHEs was measured using a four-point probe (M4P 205 system), where the four electrodes were each pitched at 1 mm at the center of the SHE. The thickness effect was not considered due to the narrow thickness of the SHE (0.20 mm).

The PTC resistance ratios (RT/R25) of the PTC-SHEs was evaluated under increasing temperature, where RT and R25 represent the resistance at a specific temperature and 25 °C, respectively. The screen-printed SHEs and PTC ink screen-printed on glass were used for the PTC ratio measurements, where the temperature of the sample was increased using a hot plate.

#### 2.3.3. Heating Behavior

The heating performance was evaluated based on the heating rate, final temperature, change in resistance, and change in power consumption from a direct current (DC) power supply (0–32 V, 6 A; PWS2326 DC Power Supply, Tektronix, Beaverton, OR, USA). The surface temperature of the SHTs was measured using a noncontact thermal imaging camera (FLIR, MSX Technology, Wilsonville, OR, USA) while power was applied. The resistance and power consumption were measured once no temperature change was observed after a specific voltage was applied to the SHT for 10 min. The heating behavior of the SHTs in a low-temperature environment was evaluated based on the final temperature reached within an external temperature of 5 °C.

#### 2.3.4. Durability against Stretching-Contraction

The SHTs were repeatedly stretched by increasing the initial length by 20% at a speed of 1200 mm/min. The heat generation durability was evaluated at DC voltages between 0 and 5.0 V, where the surface temperature of the SHTs was measured at various intervals during the stretching–contraction cycling using a thermal imaging camera.

#### 2.3.5. Durability against Washing

Washing durability was measured by comparing the heating temperatures of the SHTs before and after washing for 30 min using the wool washing mode of a mechanical washing machine (SWM-M3ET, Shinil, Korea), followed by natural drying.

#### 2.3.6. Skin Temperature Change

The practical applicability of the SHTs was verified by measuring the change in human skin temperature due to contact with the SHT. The SHT was attached under the elbow without using auxiliary means (e.g., adhesive tape or elastic band), and the skin temperature was measured before attachment, during attachment, and after detachment. Measurements for each were conducted at 1 min intervals for 20 min to determine the minimum, maximum and average skin temperatures, where the external temperature was 25 °C. An area centered on the back of the hand (radius = 4 cm) was used as the measurement point as it offers high human heat sensation and temperature change detection. A fixed power of 4.5 W was applied to the SHT, thus an SHT temperature of 51.1 °C was expected. However, the measured temperature was slightly lower because the skin acted as a heat sink for the generated heat. The temperature of the back of the hand was not affected by daily movements, thus simple movements such as the use of a mobile phone or computer were allowed during the 60 min experiment.

## 3. Results and Discussion

**Figure 6:**
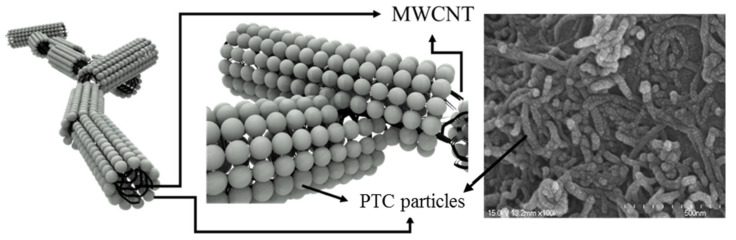
SEM images of the PTC-SHE screen-printed twice with 0.25 wt% multiwall carbon nanotube (MWCNT) ink, and illustrations of the PTC-MWCNT particles in the ink comprising PTC particles attached to the outer walls of the MWCNTs.

The surface of the PTC-SHTs was evaluated using SEM, and the PTC-MWCNT particles connected end to end on the PTC-SHT surface were illustrated (Figure 6). The SEM micrographs revealed that the PTC-MWCNT particles comprised spherical PTC particles attached to the surface of the MWCNTs. The resulting shape of the PTC-MWCNT particles was a one-dimensional linear shape with a surface covered by spherical PTC particles. The morphology of the PTC-MWCNT particles facilitated contact on the surface of the particle, and at the end regions. Thus, the measured resistance of the PTC-SHT was not attributed to contact between the wire and the MWCNTs, but instead to contact with the PTC particles on the surface. Specifically, the MWCNTs simply carried the current through the PTC particles in the wiring, as the electrical connections inside the PTC-SHT were due to the connections with the PTC particles and not due to direct contact between the MWCNTs. In addition, contact at the end regions of the particles had a greater influence on the change in resistance of the PTC-SHTs compared to surface contact that occurred when there was stretching–contraction or expansion due to temperature change.

**Figure 7:**
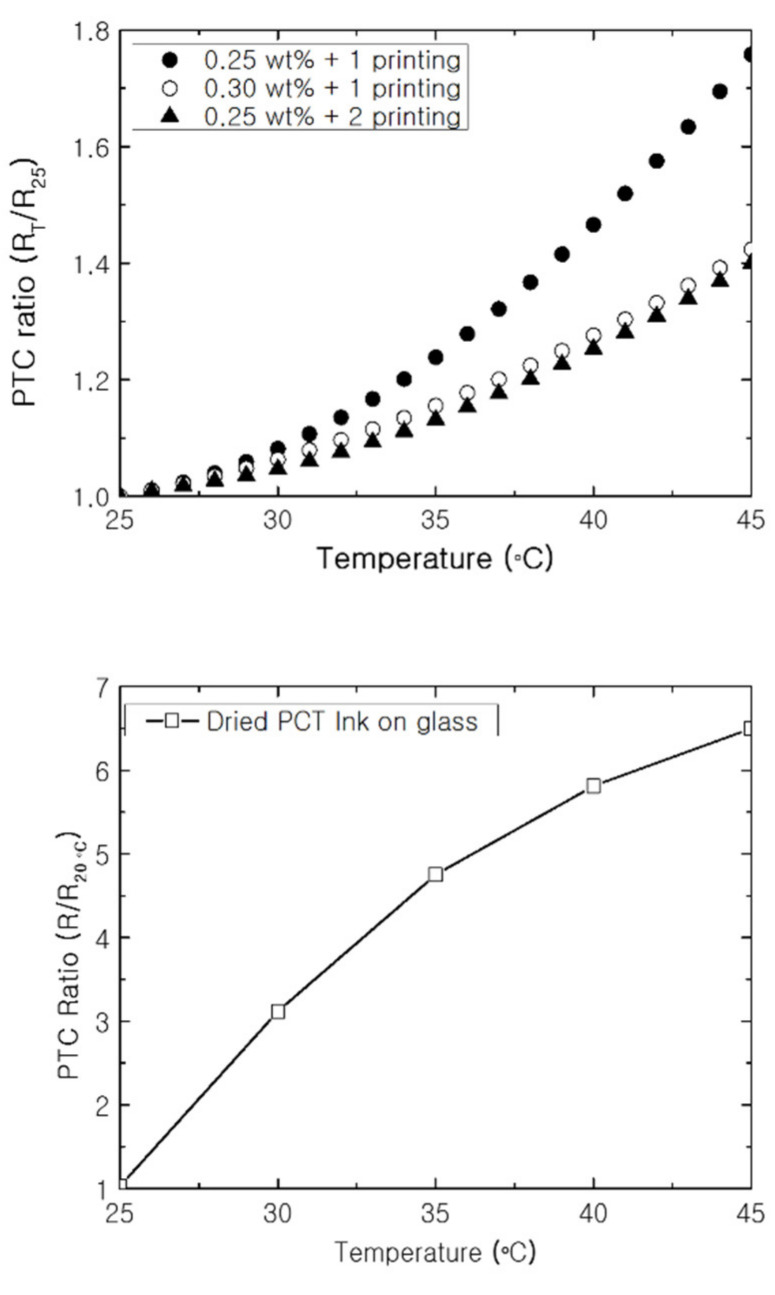
PTC ratio according to temperature increase of the PTC-SHT (**top**) and PTC ink on a glass substrate (**bottom**).

The initial resistance of the PTC-SHEs at 25 °C varied depending on the MWCNT content of the PTC paste (0.25 or 0.3 wt%) and the number of screen-printing cycles (Table 1). Further, the resistance of the NTC-SHE was 14.9 Ω-cm at 25 °C. The change in PTC ratio (R_T_/R_25_) resistance of the PTC-SHEs was evaluated as the temperature was increased from 25 to 45 °C (Figure 7). Screen-printed SHEs with varying MWCNT contents and numbers of screen-printing cycles and PTC ink screen-printed on a glass substrate were investigated. All of the PTC-SHTs exhibited typical PTC properties regardless of MWCNT content and number of screen-printing cycles.

The PTC-SHE screen-printed once with 0.25 wt% MWCNTs exhibited an initial resistance of 5.05 Ω-cm. Further, the current was lower than that of the PTC-SHTs manufactured under the other two conditions at the same voltage, which prevented rapid heating from a low temperature to a desired temperature. The PTC-SHE screen-printed once with 0.3 wt% MWCNTs ink exhibited an initial resistance of 4.35 Ω-cm, but microcracks were observed under an optical microscope and with the naked eye. This microstructure increased the likelihood of peeling or crushing during use, stretching, and washing. As the SHE screen-printed twice with 0.25 wt% MWCNTs did not exhibit microcracks, the resistance increased by 39% as the surface temperature was increased from 25 to 45 °C. Thus, the heat generation characteristics, elasticity durability, washing durability, and heat generation behavior in a low-temperature environment were evaluated using the PTC-SHT screen-printed twice using PTC paste comprising 0.25 wt% MWCNTs.

**Figure 8:**
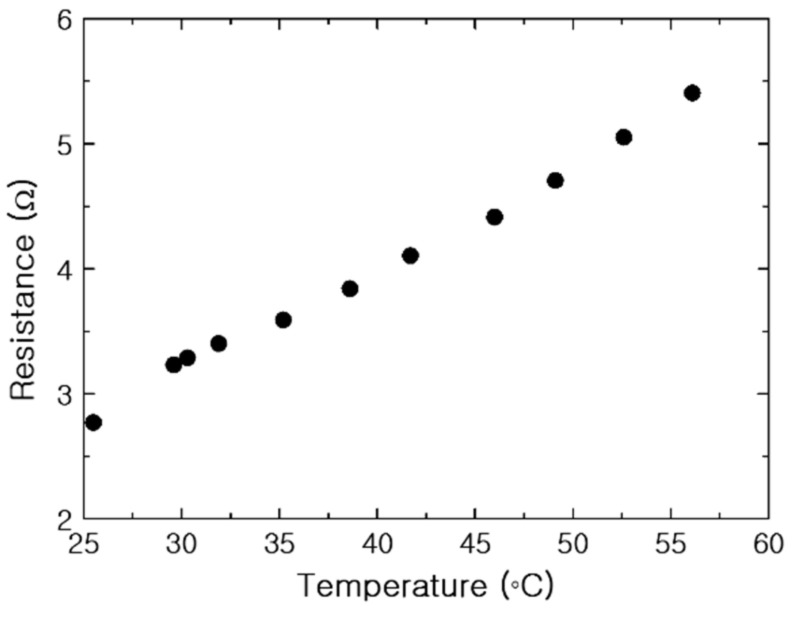
Resistance of the PTC-SHT according to temperature.

The resistance of the PTC-SHT increased with increasing temperature when the applied voltage was increased by 0.5 V at each interval (Figure 8). A nonlinear increase in resistance was observed at the beginning of voltage application with increasing temperature, while the linear relationship typically associated with PTC characteristics was observed from temperatures above 32 °C. Increases in temperature after voltage application led to simultaneous changes in the PTC value and the contact state between the PTC-MWCNT particles. Ideally, the change in resistance that occurs soon after initial voltage application is only attributed to PTC characteristics according to temperature change. However, it can also be caused by changes in the contact state between the PTC-MWCNT particles. The change in contact state at the ends of the PTC-MWCNT particles occurred predominantly in the initial temperature increase stage, and the effect subsided as the temperature continued to increase (Figure 8). Therefore, the nonlinear relationship between resistance and temperature from room temperature to 32 °C was related to two factors—namely, the small change in the contact state between the PTC-MWCNT particles and the PTC characteristics, while the linear relationship above 32 °C was attributed to the PTC effect alone (Figure 8). This linear relationship suggested that the temperature of the PTC-SHT can be controlled using a very simple temperature control circuit, which is one of the most desirable characteristics for practical application as a functional warm clothing material.

**Figure 9:**
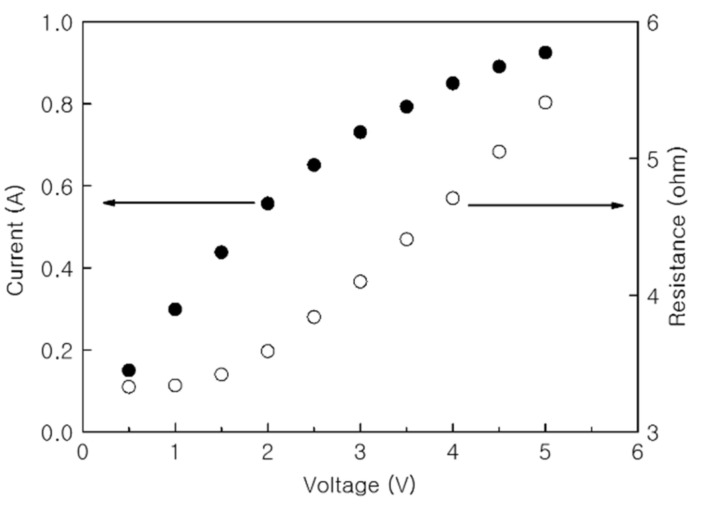
Current and resistance of the PTC-SHT according to applied voltage.

The increase in current and resistance of the PTC-SHT according to applied voltage was evaluated (Figure 9). The resistance increased relatively linearly throughout the applied voltage range, although a partially nonlinear resistance increase was observed below an applied voltage of 1.5 V (Figure 9). Unlike resistance, the current increased linearly as the voltage was initially applied, but the rate of increase dropped off above 2.5 V. As discussed, the internal resistance of the MWCNTs decreased during the initial rise in temperature, where PTC behavior became dominant at the contact points between the PTC-MWCNT particles above 32 °C. As the PTC characteristics became dominant, the total current flowing through the MWCNTs had a more dominant effect on the total current flowing through the PTC-SHT compared to the change in the low resistance of the MWCNTs.

**Figure 10:**
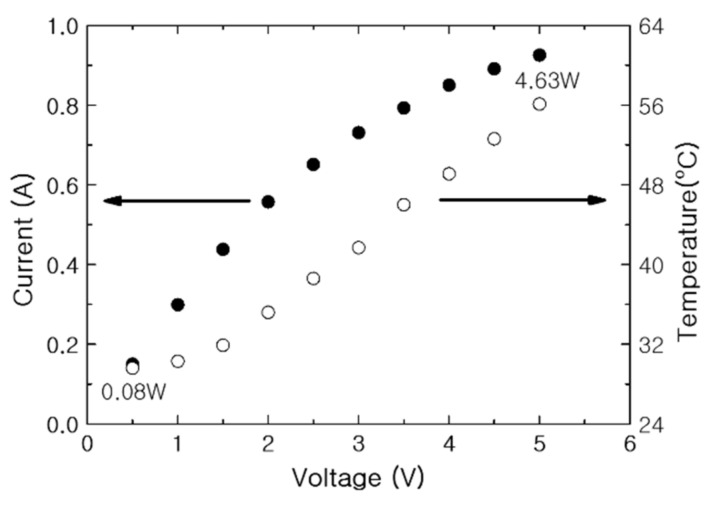
Current and temperature of the PTC-SHT according to applied voltage.

The heating temperature and current of the PTC-SHT were evaluated according to applied voltage (Figure 10). The temperature increased almost completely linearly above heating temperatures of 32 °C, while the rate of increase in the current decreased to slightly above 2.5 V. This behavior suggested that the PTC-SHT composed of PTC-MWCNT composite particles exhibited typical PTC heating behavior within the applied voltage range of 1.5 to 5 V.

**Figure 11:**
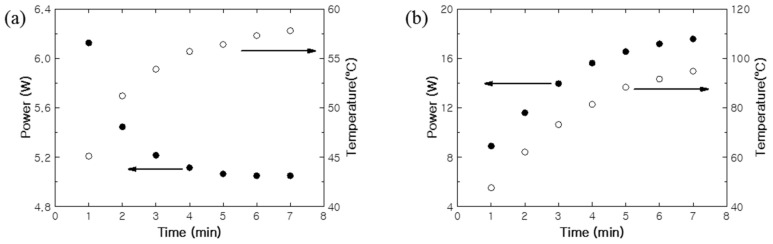
Power consumption and heating behavior of the (**a**) PTC-SHT and (**b**) negative temperature coefficient (NTC)-SHT under DC voltage of 5 V.

The power consumption and average heating temperature over time were determined by measuring the current and heating temperature at 1 min intervals while applying a constant DC voltage of 5 V to the PTC- and NTC-SHTs (Figure 11). The PTC-SHT exhibited a low initial resistance at room temperature and reached an average heating temperature of 45.1 °C after the 1 min, while the NTC-SHT reached 47.6 °C. The currents applied to the PTC- and NTC-SHT were 1.23 and 1.78 A, respectively, while 6.13 and 8.9 W were consumed, respectively, after 1 min. The temperature increase in the PTC-SHT gradually slowed from the rapid initial heating after 3 min, and an almost constant power consumption for heat generation was maintained after 6 min. Consequently, a relatively stable average heating temperature of 56.1 °C was maintained after 7 min. Thus, the heating element rose to a certain degree, and the power consumption was reduced. On the contrary, the NTC-SHT exhibited gradual increases in both power consumption and heating temperature over time. Thus, a precise and complex temperature control circuit system is required to maintain a specific operating temperature when the NTC-SHT is used. In addition, the NTC-SHT consumed ~30% more power than the PTC-SHT, even at a heating temperature of only 60 °C. Overall, the PTC-SHT can be used for ~30% longer than the NTC-SHT at a heating temperature of 60 °C using a battery with the same capacity, and a simpler temperature control circuit can be used. Further, the PTC-SHT offers superior safety, as low-temperature burns are less likely due to the minimized risk of overheating. These findings clearly demonstrate that the PTC-SHT is more suitable for applications in warming clothing, as constant temperature heating can be achieved for a long time due to low power consumption and no abnormal overheating.

The PTC-SHT maintained a constant temperature after applying a DC voltage of 5 V for 7 min or more. In addition, the temperature of the PTC-SHT was measured using thermal imaging at applied voltages of 2, 3, 4, and 5 V DC for 8 min to determine the heating temperatures achieved at the various applied voltages. Although the difference between maximum and average heating temperature gradually increased with increasing applied voltage, this difference remained below 2.8% throughout the entire applied voltage range. This demonstrated that various set heating temperatures can be maintained, where the desired temperature of the heated clothing can be achieved and maintained using a simple temperature controller that adjusts the applied voltage.

**Figure 12:**
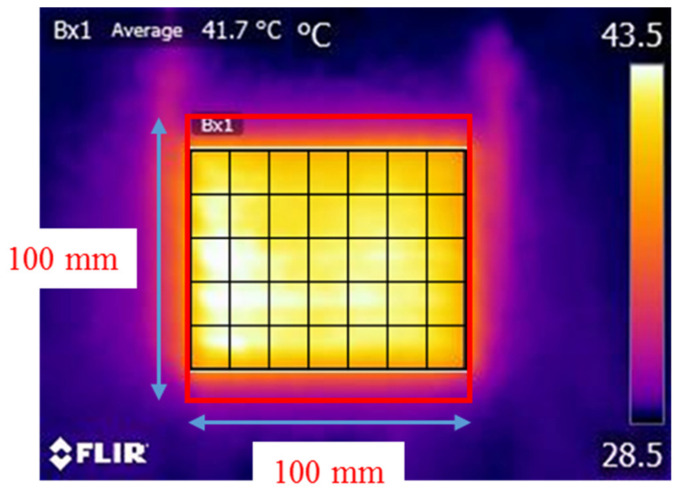
Heating temperature uniformity across the PTC-SHT at an applied voltage of 3 V.

The heating temperature uniformity across the entire PTC-SHT was evaluated by dividing the area of the sample into 35 zones and calculating the difference between the temperature of each zone and the average temperature of the PTC-SHT (Figure 12 and Table 2). All 35 zones exhibited uniform temperatures within ±2 °C of the average temperature (41.7 °C). The heating uniformity was expressed as a ratio of temperature deviation using two different methods:

(1) $$\mathrm{Uniformity}\left(\%\right)=\;\frac{{\mathrm{Temp}}_{\max}-{\;\mathrm{Temp}}_{\min}}{{\mathrm{Temp}}_{\mathrm{avg}}+{\;\mathrm{Temp}}_{\min}\;}\;\times \;100\left(\%\right)$$

(2) $$\mathrm{Uniformity}\left(\%\right)=\;\frac{{\mathrm{Temp}}_{\mathrm{std}}}{{\mathrm{Temp}}_{\mathrm{avg}}}\;\times \;100\left(\%\right)\;$$

Equation (1) is a general method for calculating uniformity based on the maximum and minimum temperatures and is mainly applied when the measurement area is small. Equation (2) is based on the mean and standard deviation, which can be applied when there are many measurement areas. The heating uniformity values according to Equations (1) and (2) were 4.2 and 0.2%, respectively.

**Figure 13:**
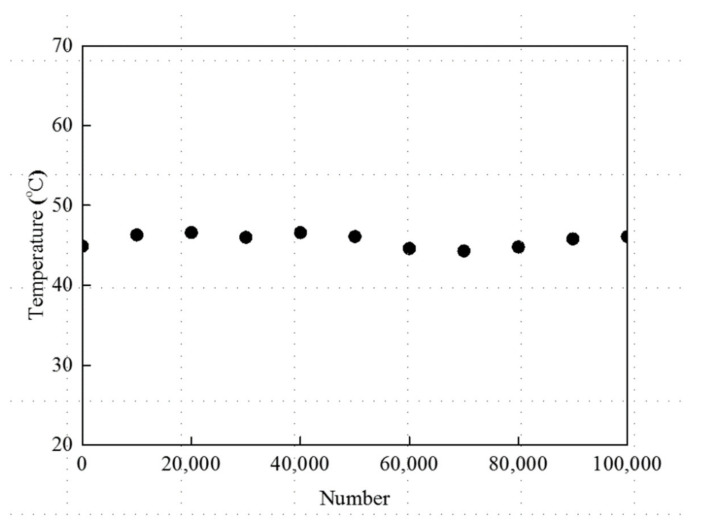
Heating temperature of the PTC-SHT at an applied voltage of 3V during stretching–contraction cycling.

**Figure 14:**
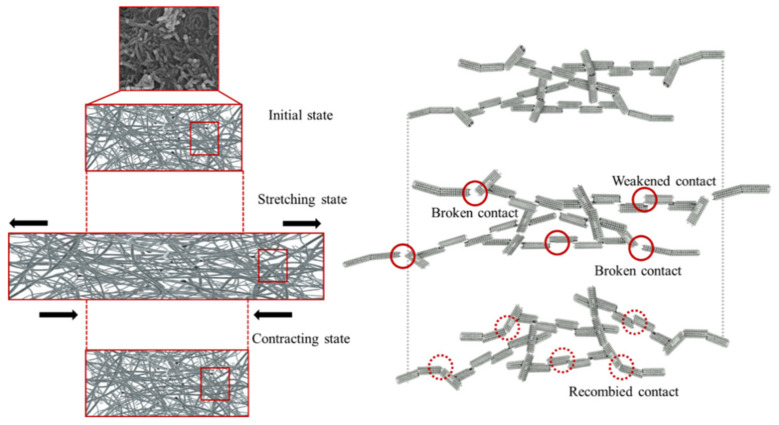
Schematic diagram of the changes in the contact state between the PTC-MWCNT particles during repeated stretching–contraction.

The use of the PTC-SHT in clothing is expected to involve repeated stretching and contractions according to human movement. Therefore, the durability of the heat generation characteristics over a large number of stretching–contraction cycles is a very important performance parameter. Thus, the changes in resistance and heating temperature of the PTC-SHT according to the applied voltage and power were evaluated during prolonged stretching–contraction cycling. The heating temperature of the PTC-SHT after 8 min at an applied voltage of 3 V fluctuated slightly between 20,000 and 100,000 cycles (Figure 13) but only deviated 1.3 to 3.8% from the initial value. These small changes in heating temperature were attributed to changes in the contact state between the PTC-MWCNT particles during stretching–contraction cycling, which altered the contact resistance (Figure 14). The contact state between the PTC-MWCNT particles was the same as that in the initial state but stretching of the PTC-SHT led to a weakened bonded state and generated contact defects such as a broken bonded states. These contact defects were limited by the thermal transfer TPU and the generated contact defects were recombined due to the elastic restoring forces of the TPU upon PTC-SHT contraction. However, the state of contact between the particles after restoration did not return completely to the initial state. Therefore, a slight change in the resistance of the PTC-SHT is expected due to stretching and contraction during practical use, where continued changes in resistance will gradually lead to differences in heating temperature. However, this change in heating temperature due to stretching–contraction was minimal after 10,000,000 cycles, thereby indicating that no bonds were irreversibly broken. This suppression of PTC-MWCNT particle rearrangement was attributed to the highly elastic TPU thermally attached to the top and bottom surfaces of the PTC-SHT. Overall, stable heating behavior was maintained by minimizing the change in resistance of the SHE, where the changes in the physical contact state between the PTC-MWCNT particles due to repeated stretching–contraction were not large enough to change the heating behavior. Thus, deterioration of the warm functions of the PTC-SHT is not expected, even if it is deformed during use.

**Figure 15:**
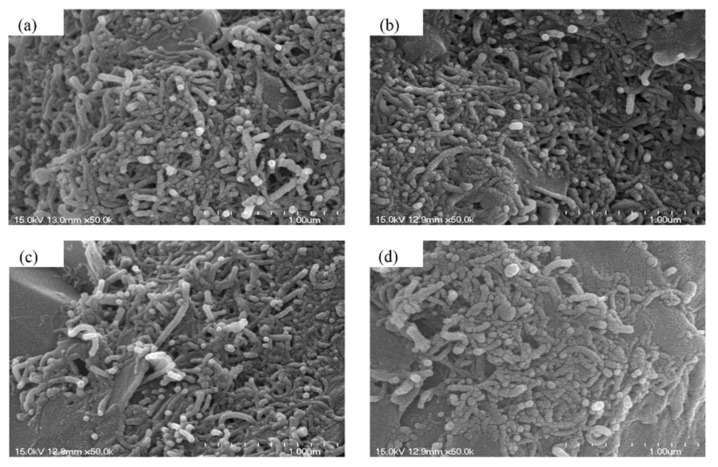
SEM images of the PTC-SHT after an applied voltage of 5 V for (**a**) 24, (**b**) 48, (**c**) 96, and (**d**) 240 h after 40,000 stretching–contraction cycles.

After 40,000 stretching–contraction cycles, 5 V was applied to the PTC-SHT and the heating conditions were maintained for 24, 48, 96, and 240 h. The structural characteristics of the PTC-SHT were observed at a rate of 50,000 times using SEM. The applied voltage of 5 V corresponded to a power consumption of 4.63 W, where the PTC-SHT maintained a temperature of ~55 °C after 7 min of voltage application (Figure 10 and Figure 11). No microstructural changes were observed after continuous use (Figure 15). Further, the heating temperature of ~55 °C was maintained during continuous operation for 24 to 240 h at 4.63 W power after 40,000 stretching–contraction cycles. Thus, the PTC-SHT is expected to maintain constant heating characteristics when powered by a continuous direct current of 5 V from a typical portable battery. Further, the actual power consumption is expected to be less than 4.63 W because the power consumption required to achieve a heating temperature of 55 °C will decrease over time. Thus, the PTC-SHT can stably maintain its warm characteristics for a prolonged time, even with low power consumption and when folded or wrinkled during practical use. These characteristics are highly suitable for functional warm clothing.

**Figure 16:**
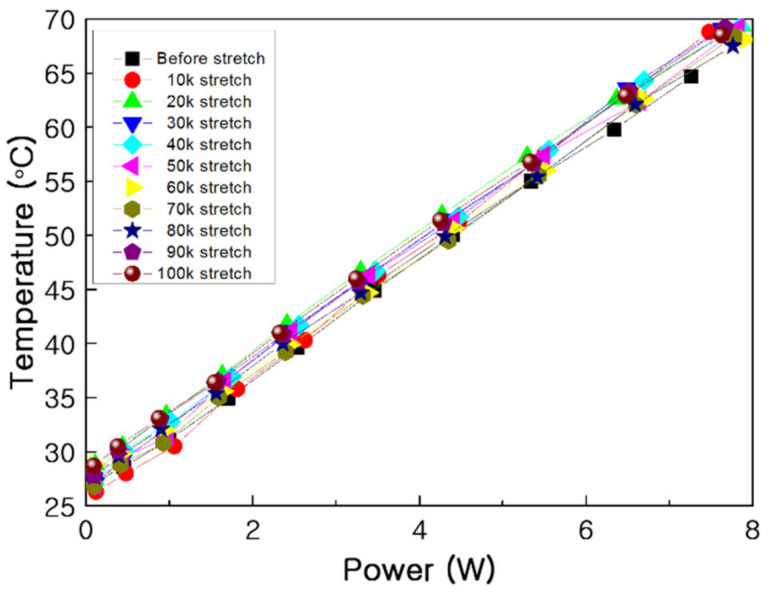
Power consumption and average heating temperature during stretching–contraction cycling.

Stretching–contraction cycling was performed for 100,000 cycles and the effect of stretching–contraction on the heating temperature according to the power consumption was evaluated under contraction at intervals of 10,000 cycles (Figure 16). The difference in heating temperature of below 5% demonstrated the high heat generation reproducibility of the PTC-SHT. This was attributed to fine rearrangement of the PTC-MWCNT particles after repeated stretching–contraction (Figure 14).

Despite the constant average heating temperature, a defect such as a crack or a severe short between the PTC-MWCNT particles due to stretching–contraction could lead to extremely uneven temperature distribution. The generation of these defects was investigated by determining the heating temperature uniformity after stretching–contraction cycling. A voltage of 3 V was applied to the PTC-SHT for 8 min after 10,000 stretching–contraction cycles, and the heating temperature in each of the 35 zones was measured. The average temperature was 41.0 °C, which was 0.7 °C lower than that before stretching–contraction but the heating temperature of all 35 zones was within ± 2 °C of the average temperature. Thus, the heat temperature uniformity was the same before and after stretching–contraction cycling. These results suggested that no structural defects such as cracks or severe shorts between the PTC-MWCNT particles can cause significant changes in the resistance of the PTC-SHT were expected to form due to repeated stretching–contraction cycles up to 100,000 times.

**Figure 17:**
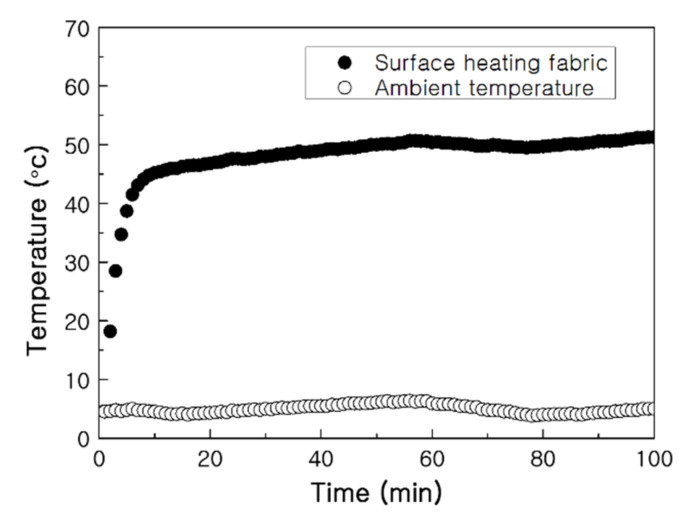
Heating behavior of the PTC-SHT at an applied voltage of 5 V from an initial temperature of 5 °C.

The applicability of the PTC-SHT in low-temperature conditions, such as winter weather, was demonstrated by evaluating the heating behavior at 5 °C. A DC voltage of 5 V was applied, and the surface temperature of the PTC-SHT was measured at 1 min intervals using a contact method with a k-type thermocouple (Figure 17). The temperature rapidly increased to 40 °C within 5 min and continued to heat to 45 °C within 10 min, and 50 °C within 40 min. The temperature was maintained thereafter. An applied voltage of 5 V at 25 °C led to a heating temperature of 56.1 °C (Figure 11). Thus, the temperature and heating rate of the PTC-SHT were 6 °C lower and slower, respectively, in a 5 °C environment. However, the SHT would typically be used inside the functional clothing and be under the influence of the user’s body temperature, thus application of the PTC-SHT in functional clothing is expected to offer a sufficient warming function to maintain body temperature at subzero temperatures.

**Figure 18:**
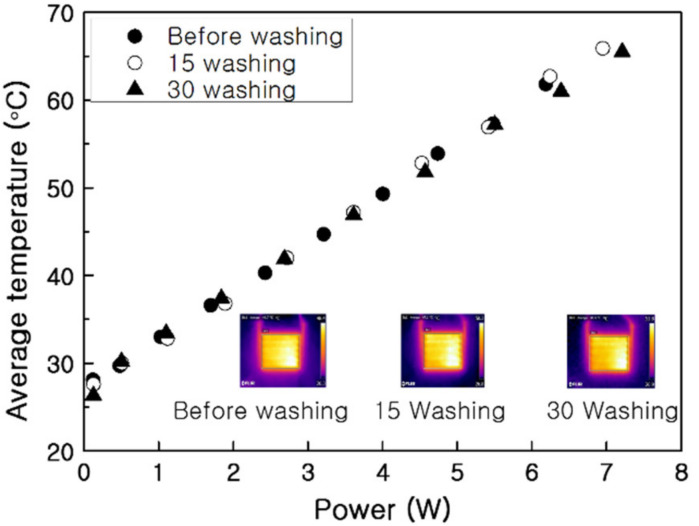
Heating temperature of the PTC-SHT according to power consumption before and after washing (inserts: thermal images at an applied voltage of 3 V).

The biggest drawback of functional clothing with electrical components is loss of function or performance degradation of the installed device due to washing. The effect of washing on the heating behavior of the PTC-SHT was evaluated based on the average heating temperature according to power consumption in an unwashed sample and samples were washed 15 and 30 times (Figure 18). The average heating temperature increased linearly with increasing power consumption and was well-maintained even after 30 washes. In addition, the heating temperature increased to the same level (each slope) in all three cases, depending on the amount of power applied. This indicated no damage or large three-dimensional defects were formed in the PTC-SHT during washing. Thus, the high durability of the PTC-SHT against washing was demonstrated.

**Figure 19:**
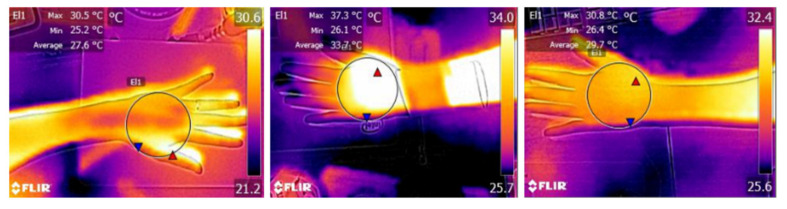
Thermal image of the back of the hand before (**left**), during (**middle**), and after (**right**) SHT attachment.

The best way to demonstrate the warming effect of PTC-SHT for maintaining human body temperature is measuring the temperature change of the human skin in contact with the PTC-SHT. The average skin temperature before PTC-SHT attachment was 27.6 °C, and increased by 6.1 °C during attachment to achieve an average temperature of 33.7 °C (Figure 19). The thermal images clearly demonstrated that simply placing the PTC-SHT on the skin increased the skin temperature rapidly. The average skin temperature decreased to 29.7 °C within 20 min of removing the PTC-SHT.

## 4. Conclusions

A paste exhibiting PTC heating behavior was prepared via a very simple mechanical mixing method and was simply screen-printed on a high-stretch fabric to produce a PTC-SHT. A highly elastic TPU film was used as external layers on the top and bottom of the PTC-SHT to maximize its durability against stretching–contraction and washing. The microstructure of the PTC-SHT comprised PTC particles attached to the surface of MWCNTs to form PTC-MWCNT particles that were connected end to end. Typical PTC heating behavior was observed, where the internal resistance of the heating element increased with increasing temperature under an applied voltage. Although MWCNTs typically exhibit NTC characteristics, where the resistance decreases with increasing temperature, the SHEs based on PTC-MWCNTs exhibited typical PTC characteristics because the resistance characteristics were determined by the PTC particles surrounding the MWCNTs, not by the MWCNTs themselves. This PTC behavior prevented excessive power consumption of the heating element that typically occurs in NTC-SHTs as heating occurs, and also reduced the power consumption after the target temperature was reached. The heating rate of the PTC-SHT upon the application of power was much faster than that of the NTC-SHT, and the target temperature was maintained once reached. In contrast, the NTC-SHT exhibited a continuous increase in power consumption and heating temperature. Further, the NTC-SHT required more power to achieve a target temperature than the PTC-SHT. Thus, functional clothing based on the PTC-SHT would function for a longer period of time than clothing with the NTC-SHT, assuming that the same batteries were used. Simply placing the PTC-SHT on the elbow area of the item of clothing, with an external temperature of 25 °C, increased the temperature of the sensitive hand area by 6.1 °C. This suggests that an SHT with PTC characteristics is very effective in maintaining body temperature in parts of the body that are very sensitive to cold external temperatures and shows promise as application in clothing with a stable warm functionality.

## Figures and Tables

**Table 1:** PTC-SHE resistance according to MWCNT contents and number of screen-printing cycles.

wt% MWCNTs	Screen-Printing Cycles	Average Resistance (Ω-cm at 25 °C)
0.25	1	5.05
0.25	2	2.66
0.3	1	4.35

**Table 2:** Deviation of the measured temperature in each zone from the average temperature of the PTC-SHT (41.7 °C).

Measured Temperature (°C)						
41.0	41.0	40.5	40.5	40.6	40.5	39.7
42.3	42.3	41.7	41.7	42.1	42.0	40.5
42.8	42.9	42.3	42.3	42.6	42.5	41.1
42.5	43.2	42.8	42.5	42.9	42.6	41.2
42.2	43.0	42.6	42.4	42.6	42.4	40.9
**Temperature Deviation from Average Temperature (°C)**						
−0.7	−0.7	−1.2	−1.2	−1.1	−1.2	−2.0
0.6	0.6	0.0	0.0	0.4	0.3	−1.2
1.1	1.2	0.6	0.6	0.9	0.8	−0.6
0.8	1.5	1.1	0.8	1.2	0.9	−0.5
0.5	1.3	0.9	0.7	0.9	0.7	−0.8

## Data Availability

Not applicable.
